# Autobiographical Memory and Psychological Distress in a Sample of Upper-Limb Amputees

**DOI:** 10.1371/journal.pone.0099803

**Published:** 2014-06-12

**Authors:** Martina Luchetti, Ornella Montebarocci, Nicolino Rossi, Andrea G. Cutti, Angelina R. Sutin

**Affiliations:** 1 Department of Psychology, University of Bologna, Italy; 2 I.N.A.I.L., Prosthesis Centre, Vigorso di Budrio, Bologna, Italy; 3 Department Behavioral Sciences and Social Medicine, Florida State University College of Medicine, Florida, United States of America; Harvard Medical School, United States of America

## Abstract

Amputation is a traumatic and life-changing event that can take years to adjust to. The present study (a) examines psychological adjustment in a specific trauma-exposed sample, (b) compares the phenomenology (e.g., vividness) of amputation-related memories to more recent memories, and (c) tests whether memory phenomenology is associated with psychological distress. A total of 24 upper-limb amputees recalled two autobiographical memories–an amputation-related memory and a recent memory–and rated the phenomenological qualities of each memory, including Vividness, Coherence, Emotional Intensity, Visual Perspective, and Distancing. Participants also completed self-rated measures of psychological distress and personality. The sample was generally well adjusted; participants showed no relevant symptoms of anxiety and depression, and personality scores were similar to the general population. There were no significant differences in phenomenology between the two types of memories recalled. Even though amputation-related memories were, on average, almost 20 years older than the recent memories, they retained their intense phenomenology. Despite the intensity of the memory, none of the phenomenological dimensions were associated with psychological distress. It is worth to further define which dimensions of phenomenology characterize memories of traumatic events, and their association with individuals' psychological reactions.

## Introduction

Amputation is a life-changing event that has widespread implications for psychological and social functioning. Amputees tend to have higher rates of depression than non-clinical samples (e.g., [1]–[4]; see [5]) and report less life satisfaction than healthy controls [6]. Although symptoms of distress are relatively comparable between upper-limb and lower-limb cases [3], major depression and post-traumatic stress symptoms may be greater among upper-limb amputees [7]. Upper-limb amputation differs from lower-limb amputation in terms of cause and pattern of comorbidities. It is more likely to be due to traumatic injury and to occur in relatively young and healthy adults [8]. Despite the potentially greater impact of upper-limb amputation [9], less research has addressed this group of amputees compared to lower-limb amputations. Given the personal and social importance of the hand and arm, amputation of an upper limb can be a particularly traumatic event.

There is a great need to identify how psychosocial factors contribute to the post-amputation adjustment process [10], . Psychological reactions to life threatening experiences, such as physical illness or injuries, can impede adjustment and prolong the effects of the trauma. In particular, the way people retrieve memories of traumatic experiences may be a further source of psychological distress [13]–[15]. Autobiographical memories differ across various phenomenological qualities, such as the specificity of the retrieved autobiographical information, the re-experience of vivid details and images, and the intensity of emotions evoked during recall [16], [17], and these qualities have been linked to psychological functioning [18]. Thus, even memories with similar content may have very different phenomenological qualities. Memories constitute significant material to work on during the flow of clinical interactions [19], [20]. Working with trauma-related memories (e.g., writing about trauma-related feelings and thoughts [21]) may improve psychological, social and biological functioning [22]. Few studies, however, have examined memory features in specific medical populations (e.g., cancer patients [23]–[25]), and, to our knowledge, no study has examined autobiographical memories in amputees.

The present study examined memory phenomenology and psychological functioning in a sample of upper-limb amputees. Specifically, we (a) examined the long-term state and trait psychological adjustment of upper-limb amputees, (b) tested whether there were differences in phenomenology between an amputation-related and a recent non-amputation-related memory, and (c) tested whether memory phenomenology was associated with psychological functioning. We consider the amputation-related memory to be a memory of a trauma (i.e., “a body wound or shock produced by sudden physical injury, as from violence or accident”), which is not necessarily equivalent to a traumatic memory (i.e., a memory that produces psychological distress). As such, a memory of a trauma such as amputation may or may not also be considered a traumatic memory.

## Methods

### Ethics statement

Ethical approval for the study was obtained from the ethical committee of the University of Bologna and institutional review board of the Florida State University. Written informed consent was obtained by all participants.

### Procedure and participants

A total of 24 upper-limb amputees referring to the prosthetic center of the Italian Workers' Compensation Authority (INAIL, Vigorso di Budrio, Bologna, Italy) participated in this study. All participants were male and had a work-related traumatic amputation. Participants were, on average, 52.7 years old (SD = 12.6; range 24–71; median 53.5). The average time elapsed since the amputation was 19.3 years (SD = 13.9; range 0.6–48.9; median 18). Most participants had a high school education (79.2%), were married (75.0%), and employed (41.7%). All amputees used a prosthesis (95.8% myoelectric prosthesis) for more than 8 h/day.

Participants completed measures of personality and psychological distress, and retrieved and rated two autobiographical memories. The measures and memories were counterbalanced across participants, as were the order of the memory requests; no order effects were detected.

### Measures

#### Psychological functioning

Participants completed the Hospital Anxiety and Depression Scale (HADS[26]), the Impact of Event Scale (IES[27]) and the Big Five Inventory (BFI[28]). The HADS is a 14-item self-report questionnaire specifically designed to recognize anxiety and depression in medical patients. The IES is a 15-item questionnaire that measures avoidance and intrusion experiences that reflect the intensity of post-traumatic stress. The BFI is a 44-item questionnaire of the five broad dimensions of personality: extraversion, agreeableness, conscientiousness, neuroticism, and openness.

#### Memory task

Participants retrieved two autobiographical memories: one related to their amputation and one of a recent event. For the amputation memory, participants recalled a specific memory related to their amputation. Participants described the accident that led to the amputation (n = 8), the amputation surgery that followed the accident (n = 7), or the sequence of events between the accident and the surgical intervention (n = 9). For the recent memory, participants recalled a memory of an event that occurred within the last 1–2 years, without constraint on valence or content. For each memory, participants reported the time elapsed since the event occurred. The memory narratives were audiotaped, taking note of duration time.

#### Memory phenomenology

Participants completed three scales of the Memory Experiences Questionnaire (MEQ[17]): Vividness (e.g., “My memory for this event is very vivid”), Coherence (“This memory is of an event that occurred once at a particular time and place, not a summary or merging of many similar or related events”), and Emotional Intensity (“My emotions are very intense concerning this event”). All scales included reverse-scored items to control for acquiescence. Participants also reported the visual perspective of each memory (i.e., “When you think about this memory, do you see the event from your own eyes or from the eyes of an observer?”; 1 = *from my own eyes*, 2 = *from the eyes of an observer*), and the perceived psychological distance from the event in the memory (i.e., “When you think about this memory, how different do you think you are now from the person in the memory?”; from 1 = *very similar* to 4 = *completely different*).

## Results

Descriptive statistics are shown in Table 1. On average, participants retrieved an amputation-related memory that was nearly 20 years old, whereas the recent memory was approximately 0.9 years old (Z = −4.20, *p*<.001). The narrative of the amputation memory was also longer (Z = −2.97, *p*<.01). Participants were generally well adjusted. Depression and anxiety scores were within the normal range; only 1 participant (4.2%) met the common cutoff for depression and six (25%) scored above the cutoff for anxiety. Scores on the IES subscales of Intrusion and Avoidance indicated that participants were relatively well adjusted to the trauma; only 2 participants (8.4%) reported significant post-traumatic psychological stress (total IES scores≥35). Personality scores were within the range found in non-clinical samples [29]. Time since amputation was unrelated to psychological functioning.

**Table 1:** Descriptive statistics for all study variables.

Variables	Mean (SD)	
	Amputation Memory	Recent Memory
Memory Age (years)	19.37 (13.94)	0.88 (0.44)
Narrative Duration (seconds)	198.96 (100.07)	154.79 (88.63)
Vividness	28.29 (3.08)	28.04 (2.95)
Coherence	25.58 (3.08)	25.21 (3.31)
Emotional Intensity	18.71 (3.86)	18.75 (3.31)
Observer Perspective (% 3rd perspective)	12.5	4.2
Distancing (% feeling different)	25.0	4.2
HADS Anxiety	4.96 (3.44)	
HADS Depression	2.87 (2.49)	
IES Intrusion	13.67 (5.25)	
IES Avoidance	5.33 (1.93)	
Extraversion	28.62 (5.72)	
Agreeableness	35.42 (5.24)	
Conscientiousness	34.79 (6.22)	
Neuroticism	20.37 (4.75)	
Openness	37.46 (7.29)	

N = 24. HADS  =  Hospital Anxiety and Depression Scale. IES  =  Impact of Event Scale.

The phenomenology of the amputation memory and the recent memory were quite similar (Table 1). Participants reported both memories as vivid, coherent, and emotionally intense. Despite differences in the time between the memories, no significant differences in phenomenology were observed between the amputation-related and the recent memories (*p*s>.05). Few participants retrieved their memories from the 3^rd^ person perspective (12.5% of amputation memories, 4.2% of control memories) or perceived the recalled events as psychologically distant (25.0% of amputation memories, 4.2% of control memories). The percent of 3^rd^ person and psychologically distant memories did not significantly differ across memory type.

We next examined the intercorrelations among the phenomenology dimensions and whether these associations varied by memory (Table 2). Memories rated as vivid, both the amputation and recent, were also rated as a logical story in a specific time and place (i.e., coherent). For the amputation-related memory, memories rated as coherent were also rated as emotionally intense, whereas memories retrieved from a 3^rd^ person perspective were vague, incoherent, and emotionally faded. However, none of the correlation coefficients between the two memories differed significantly from each other.

**Table 2:** Intercorrelations among memory qualities, psychological distress and personality traits.

	Vividness		Coherence		Emotional Intensity		Visual Perspective		Distancing	
	Amputation Memory	Recent Memory	Amputation Memory	Recent Memory	Amputation Memory	Recent Memory	Amputation Memory	Recent Memory	Amputation Memory	Recent Memory
Vividness	1	1	.46*	.79**	.19	.35	−.61**	−.32	−.31	−.23
Coherence			1	1	.49*	.08	−.53**	−.20	.19	−.27
Emotional Intensity					1	1	−.45*	−.20	.17	.17
Visual Perspective							1	1	.06	.12
Distancing									1	1
HADS										
Anxiety	.06	.14	−.00	.00	−.02	.22	.10	−.35	−.24	−.49*
Depression	−.31	−.10	−.04	−.25	.08	.04	.15	−.32	.06	.02
IES										
Intrusion	.11	.14	.00	.13	.07	−.08	−.33	−.29	−.06	−.00
Avoidance	−.32	−.19	−.31	−.31	−.09	.12	.17	−.18	−.12	−.26
BFI										
Extraversion	.18	−.07	.18	.05	.34	−.06	−.21	−.26	−.43*	−.19
Agreeableness	.37	.20	.05	.18	.06	.08	−.22	−.20	−.52**	.04
Conscientiousness	.06	−.24	−.26	−.33	−.30	.20	.04	.01	−.42*	−.08
Neuroticism	−.14	−.03	−.12	−.10	−.34	−.13	.24	.14	.13	−.24
Openness	.45*	.25	.30	.25	.14	.05	−.34	−.07	−.30	−.00

N = 24. HADS  =  Hospital Anxiety Depression Scale; IES  =  Impact Event Scale; BFI  =  Big Five Inventory.

**. Correlation is significant at the 0.01 level (Spearman Correlation; 2-tailed).

*. Correlation is significant at the 0.05 level (Spearman Correlation; 2-tailed).

Finally, we tested the association between memory phenomenology and participants' psychological functioning. Most associations were not statistically significant given the small sample size (*p*s>05). Open participants, however, retrieved more vivid amputation-related memories, and extraverted, conscientious, and antagonistic participants perceived the traumatic event as less psychologically distant. Despite one correlation between anxiety and distancing, phenomenology was notably unrelated to symptoms of anxiety and depression and intrusion and avoidance.

## Discussion

We examined psychological functioning and autobiographical memory phenomenology in a sample of upper-limb amputees. Patients reported relatively intense phenomenology for both the amputation-related and control memories–i.e., they rated their memories as vivid, coherent and emotionally intense–and most retrieved their memories from a 1^st^ person perspective and did not distant themselves from the memories. Notably, there were no differences in phenomenology between the two types of memory recalled, and patients showed no relevant symptoms of anxiety and depression.

The present study yielded two main findings. First, our sample was psychologically well-adjusted. Levels of anxiety and depression were lower compared to levels reported in other studies of upper-limb amputees [2],[3], and personality scores were similar to those of non-clinical samples [29]. Second, even though the amputation-related memories were much older than the recent memories, these memories showed similar characteristics. Although phenomenology tends to decline with the age of the memory [17], the amputation-related memories retained their intense phenomenological qualities, even 20 years later. In contrast to nonclinical samples that have found that phenomenologically-intense memories are associated with distress [18], the powerful phenomenology of the amputation-related memory did not foster psychological distress in the current sample.

This research has limitations that need to be taken into account when interpreting the results. First, our sample was well adjusted. Phenomenology may have stronger links to distress when the individual is actively struggling to come to terms with the amputation. These issues should thus be addressed with a clinical sample. Second, the amputation-related memories were, on average, approximately 20 years old. Previous studies have shown that the recollections of a trauma change over time and are moderated by the trajectory of post-traumatic stress symptoms [30]. As such, participants likely had time to adjust to the trauma and integrate their experience into a coherent life story. Finally, only self-report measures were employed. It is possible that the memories were internally coherent to the participant, but the narrative reported by the participant may not be coherent and logical when rated by external coders. Coded assessments of coherence may have stronger associations with psychological distress [31].

Although the sample was small, this research adds knowledge on memory phenomenology in a specific trauma-exposed population. There are two opposite views of memories of trauma [32]. One view considers such memories as highly accessible and as vivid and coherent as the event allows, whereas the other view considers them as fragmented memories that cannot be easily recalled voluntarily as a coherent narrative. The ways in which amputees re-experience their trauma likely reflect how adjusted they are to it [17]. Few studies have explored autobiographical memory in medical patients [23]–[25], and to our knowledge this is the first study that examined memory qualities in amputees. Cancer patients commonly show difficulty retrieving specific memories [24], but impairment is not necessarily associated with levels of distress [23]. The present findings support the power of trauma-related memory but not its association with trauma-related distress. Amputation memories likely ground the “current self” and the patients' “story life” and thus remain more active and accessible compared to other memories. It is important to note that amputation represents a particular experience that may not generalize to other trauma populations. Given the personal and social importance of the hand, further studies on psychosocial outcomes in upper-limb amputees are needed.
